# A first-in-human phase 1 and pharmacological study of TAS-119, a novel selective Aurora A kinase inhibitor in patients with advanced solid tumours

**DOI:** 10.1038/s41416-020-01100-3

**Published:** 2020-10-06

**Authors:** Debbie G. J. Robbrecht, Juanita Lopez, Emiliano Calvo, Xiaomin He, Hirai Hiroshi, Nital Soni, Natalie Cook, Afshin Dowlati, Angelica Fasolo, Victor Moreno, Ferry A. L. M. Eskens, Johann S. de Bono

**Affiliations:** 1 Erasmus MC Cancer Institute, Rotterdam, the Netherlands; 2 The Royal Marsden and The Institute of Cancer Research, London, UK; 3 START Madrid-Centro Integral Oncológico Clara Campal, Hospital Madrid Norte Sanchinarro, Madrid, Spain; 4 Taiho Oncology Inc, Princeton, NJ USA; 5 Taiho Pharmaceutical Co., Ltd., Tokyo, Japan; 6 Christie NHS Foundation Trust and the University of Manchester, Manchester, UK; 7 UH Cleveland Medical Center, Case Western Reserve University, Cleveland, OH USA; 8 Unit of New Drugs and Innovative Therapies Dept. Medical Oncology San Raffaele Hospital – Scientific Institute Via Olgettina, Milan, Italy; 9 START-Madrid-FJD, Hospital Fundacion Jimenez Diaz, Madrid, Spain

**Keywords:** Drug development, Drug safety

## Abstract

**Background:**

This is a first-in-human study with TAS-119, an Aurora A kinase (AurA) inhibitor.

**Methods:**

Patients with advanced, refractory, solid tumours were enrolled into 5 dose escalation cohorts (70–300 mg BID, 4 days on/3 days off, 3 out of 4 weeks or 4 out of 4 weeks). The expansion part consisted of patients with small-cell lung cancer, HER2-negative breast cancer, *MYC*-amplified/β-catenin-mutated (MT) tumours or other (basket cohort).

**Results:**

In the escalation part (*n* = 34 patients), dose-limiting toxicities were one grade 3 nausea, two grade 2 and one grade 3 ocular toxicity and a combination of fatigue, ocular toxicity and nausea in one patient (all grade 2) at dose levels of 150, 200, 250 and 300 mg, respectively. Most frequent treatment-related adverse events were fatigue (32%), diarrhoea (24%) and ocular toxicity (24%). Toxicity grade ≥3 in ≥10% of patients were diarrhoea (15%) and increased lipase (12%). The maximum tolerated dose was 250 mg BID. Due to one additional grade 1 ocular toxicity, the RP2D was set at 200 mg BID (4 days on/3 days off, 3 out of 4 weeks), which was further explored in the expansion part (*n* = 40 patients). Target inhibition in paired skin biopsies was shown.

**Conclusions:**

TAS-119 has a favourable and remarkably distinct safety profile from other AurA inhibitors.

**Clinical trial registration:**

NCT02448589.

## Background

Mitosis in cells is strictly regulated, mostly by serine/threonine kinases. The Aurora (Aur) kinase family, classified as Aur kinase A, B and C, plays an important role in mitosis, spindle assembly checkpoint and regulation of transition from G2 to M phase.^1–3^ AurA is overexpressed in cancer cell lines and is often amplified in human cancers.^4–6^ Increased AurA protein expression in cancer cells is linked to resistance to cytotoxic agents targeting the mitotic spindle checkpoint.^7,8^ Therefore, inhibiting AurA could serve as a target for the development of anticancer drugs.

Multiple Aur kinase inhibitors have been developed to date.^2,9–11^ Toxicity, in particular grade 3 (febrile) neutropenia and mucositis, has hampered the clinical application of these compounds.^11,12^ This toxicity may be, at least in part, a consequence of cross-inhibition of other kinases of these Aur kinase inhibitors. TAS-119 is a selective and orally AurA inhibitor. Pre-clinical data showed selectivity with a half-maximum inhibitory concentration (IC50) of 1.04 (±0.09 nM) for AurA and 95 (±11 nM) and 36.5 (±6.2 nM) for AurB and AurC, respectively. In addition, inhibitory activity on tropomyosin-related kinases (TRKs) (TRK-A IC50 1.46 ± 0.16 nM; TRK-B IC50 1.53 ± 0.12 nM, TRK-C IC50 1.47 ± 0.04 nM), RET (IC50: 25.8 ± 1.5 nM) and ROS (IC50: 29.3 ± 0.8 nM) was observed.^13^ Compared to Alisertib (MLN8237), the only AurA inhibitor that has progressed to evaluation in a phase 3 trial,^14^ the IC50 for AurB was 1533 nM (±1060 nM) and the inhibitory effect on TRK-A, TRK-B, TRK-C, RET and ROS were relatively high (mean inhibition of 87, 78, 91, 50 and 80%, respectively), but its clinical significance is unknown. The effect on AurC was not described.^15^ Furthermore, TAS-119 and other AurA kinase inhibitors demonstrated more potent growth inhibitory effects on cancer cells with *MYC* oncogene amplifications and/or mutations in the Wnt/β-catenin pathway.^16,17^

We conducted a first-in-human phase 1 study with TAS-119 to assess safety and tolerability and to determine the maximum-tolerated dose (MTD) and recommended phase 2 dose (RP2D). Other objectives were the assessment of pharmacokinetics (PK), pharmacodynamics (PD) and preliminary antitumour activity of TAS-119. In the expansion part of this study, patients with specific tumour types and tumours known to harbour either *MYC* oncogene amplifications or β-catenin mutations were enrolled to further explore antitumour activity.

## Methods

### Study design

This multicentre study (six centres) consisted of a dose escalation part with a 3 + 3 design to explore safety and tolerability. Patients were enrolled into predefined dose levels (DLs; 70, 150, 200, 250, 300 mg BID) utilising a 4 days on/3 days off schedule 3 out of 4 weeks (intermittent schedule). The rationale for this intermittent schedule was based on pre-clinical data showing a more favourable toxicity profile, compared to continuous dosing regimens while maintaining antitumour activity. In addition to the 4 days on/3 days off, 3 out of 4 weeks schedule, a continuous dosing (200 mg BID) schedule with the same weekly schedule (4 days on/3 days off) administered 4 out of 4 weeks, was explored in the escalation phase. A minimum of 3 patients were to be treated at each DL, and at least 6 patients were planned to be enrolled at the MTD level. The MTD was defined as the highest DL at which <33% of patients experienced a dose-limiting toxicity (DLT) during cycle 1. The RP2D was defined as a dose below or equal to the MTD based on the evaluation of all available information (tolerability in cycles after cycle 1, PK, PD or other safety information). The RP2D was used in the expansion part of this study. Based on the safety profile assessed in the dose escalation part of this study, either the intermittent (3 out of 4 weeks) or the continuous dosing schedule (4 out of 4 weeks) could be selected.

The dose escalation part of this study enrolled patients with unselected advanced solid tumours for which no standard treatment options were available. Based on the above-mentioned rationale to select for these tumours, enrolment in the expansion part of the study was restricted to patients with either any tumours with known *MYC* gene amplification or β-catenin mutation (MT) based on local testing, patients with small-cell lung cancer (SCLC), HER2-negative breast cancer, as well as to patients with other solid tumours in a basket cohort. There was no pre-screening for *MYC* gene amplified/β-catenin-mutated tumours.

The study was approved by the local ethics committees of the participating centres and was performed according to the principles defined by the Declaration of Helsinki and Good Clinical Practice guidelines. All patients gave written informed consent prior to any study-related procedure. Study initiation was in September 2014 and completion date in August 2019 (NCT02448589).

Inclusion criteria (full description are available in the Supplementary text) were age ≥18 years; Eastern Cooperative Oncology Group performance status 0–1; and adequate bone marrow, renal and hepatic function. Patients in the dose expansion part had to undergo a core tumour biopsy, as well as paired sampling of non-tumour tissue (skin biopsies), for PD assessments if considered clinically safe and appropriate (this was optional in the dose escalation part of this study). For the expansion part of this study, patients with tumours harbouring *MYC*-amplification/β-catenin mutations were selected, based on pre-clinical evidence that *MYC* amplification as well as β-catenin mutation could sensitise to Aur A inhibition.^16–18^

### Treatment, starting dose and dose escalation

TAS-119 was administered orally BID as tablets of 25 and 100 mg strength, on an empty stomach. Based on rodent toxicology data, 70 mg BID was determined as the starting dose after conversion from the severely toxic dose in 10% of exposed animals (STD10) (63 mg/kg BID) to one tenth of the human equivalent dose. Cyclic administration (4 days on/3 days off) resulted in less frequent vomiting and liquid faeces in comparison to daily administration. These data resulted in predefined dose escalation cohorts of the intermittent schedule of 70, 100, 150, 200, 250 and 300 mg BID, 4 days on/3 days off. Non-haematological DLTs consisted of grade ≥3 toxicity (excluding nausea/vomiting lasting <48 h and controlled by anti-emetic therapy, diarrhoea lasting <48 h and responsive to anti-diarrhoea medication or hypersensitivity reactions), whereas haematological DLT consisted of any grade 4 neutropenia lasting >7 days, any febrile neutropenia (documented absolute neutrophil count <1000/mm^3^) lasting >1 h, any grade 4 thrombocytopenia or grade 3 thrombocytopenia associated with bleeding and requiring blood transfusion. In addition, any grade ≥3 drug-related toxicity (excluding hypersensitivity reactions) that prevented administration of ≥80% of the assigned dose of cycle 1 or resulted in a delay of >14 days in starting cycle 2 was considered DLT.

### Pre-treatment and study evaluations

Vital sign assessment, blood cell count, serum biochemistry, coagulation parameters, urinalysis, a 12-lead electrocardiogram and, if applicable, a pregnancy test were performed at baseline. In addition, after the amendment of the protocol (Amendment 2: 31-March-2015) an ophthalmologic assessment, including visual acuity, pupil shape and pupillary reflexes, extraocular motility (eye movement) and alignment, tonometry, visual field, external examination, slit-lamp examination, and fundoscopy, was performed and repeated in all patients on days 8 and 22 during cycle 1 and on day 1 of every subsequent cycle, beginning with cycle 3. This was a consequence of ocular toxicity observed in 2 out of 26 patients until that moment.

Adverse events (AEs) at baseline and during the study were recorded and graded based on the Common Terminology Criteria for Adverse Events v4.03. Tumour measurements were done at the end of every second cycle or as per the Institutional standard of care in case of clinical indications. Response were assessed using Response Evaluation Criteria in Solid Tumours (RECIST) v1.1.^19^

Blood samples for PK analysis were collected in cycle 1, on days 1, 4 and 18 pre-dose and at 0.5, 1, 2, 3, 5, 8, and 12 h post-dose. Urine samples were collected on day 1 of cycle 1 before dosing and from 0 to 12 h after dosing. Plasma and urine concentrations of TAS-119 were determined by validated liquid chromatography–tandem mass spectrometry method. PK parameters included the peak plasma concentration (*C*_max_), time to reach maximum concentration in plasma (*T*_max_), area under the plasma concentration–time curve up to the last observable concentration (AUC_0 − last_) and up to infinity (AUC_0 − inf_), terminal phase elimination half-life (*T*_1/2_), clearance (CL/F), apparent volume of distribution (*V*_d_/F), renal clearance (CL_r_) and oral clearance (CL/F).

Blood and tissue samples were taken for PD on-target effects during mitosis of TAS-119. First, the rate of phosphorylated histone H3 (pHH3) immunohistochemistry-positive cells to total cells were measured in paired skin biopsies and paired tumour samples collected prior to first TAS-119 administration as well as after receiving TAS-119 on day 4 of cycle 1. In case of target engagement, an increase in pHH3 is expected because of cells that will stagnate in mitosis. Second, pre- and post-dose mRNA expression of genes involved in mitosis, BORA, SGOL2, KIF20A and DEPDC-1, was analysed in tissue samples by reverse transcriptase-polymerase chain reaction.

The influence of polymorphisms of *SLCO1B1* encoding the drug influx transporter OATP1B1 was examined in a blood sample obtained pre-dose on day 1 of cycle 1 for all patients during the dose escalation part. *MYC* amplification and β-catenin mutation were assessed in archival formalin-fixed paraffin-embedded tumour samples obtained after enrolment of the patient into the study.

### Statistical analysis

Planned enrolment in the dose escalation part included 18–30 evaluable patients, with 3–6 DLT evaluable patients in each DL. To further assess the feasibility as well as preliminarily efficacy of the RP2D, approximately 40 patients were planned to be enrolled into the expansion part (approximately 10–15 patients in each of the expansion cohorts). An additional 20 patients were pre-planned to be enrolled in an extension of the expansion part provided that either an overall response rate (ORR) of ≥20% for each specific indication or ≥10% for patients with *MYC*-amplified or β-catenin-mutated tumours had been observed. The addition of 20 patients in the indication, which demonstrates the most promising response, provides a reasonable number of patients (*n* = 30) to be explored.

Descriptive statistics were used to summarise safety data (AEs, vital signs and clinical laboratory results) overall response based on RECIST, PK and PD data. PK parameters were calculated by standard non-compartmental methods using Phoenix^TM^WinNonlin® (Ver 6.3 or later, Certara L.P.). Dose proportionality of TAS-119 was evaluated with a power model and a linear regression model using logarithmic values of PK parameters such as *C*_max_ and AUCs, as well as a one-way analysis of variance (ANOVA) using dose-normalised parameters such as *C*_max_, AUCs, CL/F, and apparent volume of distribution (*V*_d_/F). Student’s *t* test was used to test statistical significance of the PD data and calculate the mean ratio for AUC_0 − last_ at RP2D or MTD. Influence of SLCO1B1 genotypes on PK parameters was tested by ANOVA.

## Results

Thirty-four patients were enrolled in the dose escalation part and received at least one dose of TAS-119. Four patients did not receive ≥80% of the assigned dose in cycle 1 and were deemed unevaluable. In the expansion part, 40 patients were enrolled and received at least 1 dose of TAS-119. One patient was ongoing as of the data cut-off (β-catenin-mutated non-small-cell lung cancer). Patient baseline characteristics for the both the dose escalation and expansion population are summarised in Table 1.

**Table 1:** Baseline patient characteristics in the escalation and expansion phases.

	Escalation phase	Expansion phase
	(total *n* = 34)	(total *n* = 40)
Age (years)		
Mean (SD)	66.0 (7.65)	59.4 (12.84)
Median (range)	67.0 (42–77)	60.5 (20–79)
Gender, *n* (%)		
Male	30 (88.2)	16 (40.0)
Female	4 (11.8)	24 (60.0)
Race, *n* (%)		
Caucasian	26 (76.5)	32 (80.0)
Black or African-American	1 (2.9)	2 (5.0)
Other	2 (5.9)	6 (15.0)
Not collected	5 (14.7)	
Baseline ECOG, *n* (%)		
Score 0	6 (17.6)	14 (35.0)
Score 1	28 (82.4)	26 (65.0)
Score >1	0	0
Primary tumour type, *n* (%)		
Breast		9 (22.5)
Lung	4 (11.8)	10 (25.0)
Mesothelioma	13 (38.2)	6 (15.0)
Other solid tumour^a^	17 (50)	15 (37.5)
*MYC*-amp/B-cat-mutated tumours, *n* (%)		13 (32.5)
Number of prior regimens, *n* (%)		
0	1 (2,9)	0
1	10 (29.4)	3 (7.5)
2	11 (32.4)	8 (20.0)
>2	12 (35.3)	29 (72.5)
Prior radiation therapy, *n* (%)		
Yes	17 (50.0)	26 (65.0)
Palliative	10 (29.4)	17 (42.5)
Therapeutic	6 (17.6)	13 (32.5)
N/A	3 (8.8)	1 (2.5)
No	17 (50.0)	14 (35.0)

*SD* standard deviation, *ECOG* Eastern Cooperative Oncology Group, *N/A* not evaluable.

^a^Colorectal, renal, ovarian, pancreatic, stomach/oesophageal/GE junction, prostate, head and neck, urothelial, endometrial, metastatic (adeno-) carcinoma of unknown primary, adrenal cortical carcinoma, choroidea melanoma, metastatic neuro-endocrine carcinoma, extra-skeletal chondrosarcoma.

Briefly, 5 DLs were investigated, and the median treatment duration was 57 days (range 28–651). The median delivered dose intensity was 91% (range 33.3–101.5; Table 2). For the expansion cohort, there were 10 patients with SCLC (25.0%), 9 with HER2-negative breast cancer (22.5%), 6 with mesothelioma (15.0%) and 15 with other cancer types (37.5%). Of these, 13 had either *MYC*-amplified/β-catenin-mutated tumours. The median treatment duration was 56 days (range 28–616), and the median delivered relative dose intensity was 85% (range 47.9–100) (Table 2).

**Table 2:** Study drug administration.

	Escalation phase	Expansion phase
	(total *n* = 34)	(total *n* = 40)
Total dosage of TAS-119 (mg)		
Mean (SD)	14,947.6 (18,113.7)	14,430.0 (18,998.3)
Median	10,200.0 (range 630–100,800)	9600.0 (range 4600–105,600)
Treatment duration (days)		
Mean (SD)	102.1 (113.9)	96.9 (113.0)
Median	57.0 (range 28–651)	56.0 (range 28–616)
Cycles initiated		
Mean (SD)	3.5 (4.0)	3.4 (3.9)
Median	2.0 (range 1–23)	2.0 (range 1–22)
Cycles completed		
*n*	34	40
Mean (SD)	3.1 (4.1)	2.9 (3.9)
Median	2.0 (range 0–23)	2.0 (range 1–21)
Relative dose intensity (%)		
*n*	34	38
Mean (%) (SD)	82.7 (19.8)	85.4 (16.3)
Median (%)	91 (range 33.3–101.5)	85 (range 47.9–100)

Note: The overall treatment duration is defined as the first dose date of last cycle minus first dose date + 28. If a patient died within 28 days after the first dose day of the last cycle, the overall treatment duration is defined as death date minus first dose date + 1.

*SD* standard deviation.

### Safety and tolerability

Five patients in the dose escalation part experienced DLT during cycle 1 (Supplementary Table 1): one grade 3 serious AE (SAE) of nausea (DL 2 150 mg BID), one grade 2 AE of dry eyes (DL 2.1 200 mg BID), one patient with grade 2 AEs of fatigue, corneal epithelial microcysts and nausea (DL 2.2 250 mg BID), and 2 patients at DL 3 (300 mg BID) experienced a SAE of corneal microcysts, grade 2 and 3, respectively. All events resolved without sequelae after TAS-119 was stopped.

In all, 97% of patients experienced AEs (77% treatment related). The most frequently reported AEs in the escalation part (≥10%) were fatigue (*n* = 18, 53%), pain (*n* = 18, 53%), diarrhoea (*n* = 14, 41%), ocular symptoms (*n* = 14, 41%), cough (*n* = 10, 29%), dyspnoea (*n* = 9, 27%), decreased appetite (*n* = 9, 27%) and nausea (*n* = 7, 21%). Toxicity grade ≥3 in ≥10% of patients were diarrhoea and increased lipase. AEs defined as being treatment related (TRAE) (≥10%) were fatigue (*n* = 11, 32%), diarrhoea (*n* = 8, 24%), ocular symptoms (*n* = 8, 24%), nausea (*n* = 5, 15%) and decreased appetite (*n* = 4, 12%) (Table 3). White blood cell count decrease was observed in only one patient, and mucositis or stomatitis were observed in only three patients. SAEs and treatment-related SAEs were reported for 38 and 15% of patients, respectively. Four (12%) patients had AEs leading to discontinuation of study drug; 3 (9%) were considered treatment related. One (3%) patient at DL 2 (150 mg BID) experienced an AE with the outcome of death. This patient developed disease progression, which was initially reported as a grade 3 SAE that worsened in intensity to grade 5 on the day of death. The sequelae in this patient were deemed not to be study treatment related.

**Table 3:** AEs and treatment-related AEs occurring in ≥10% of all patients, by grade.

AE	Escalation phase			Expansion phase		
	(total *n* = 34)			(total *n* = 40)		
	Any grade (*n*, %)	Treatment-related, any grade (*n*, %)	Grade ≥3 (*n*, %)	Any grade (*n*, %)	Treatment-related, any grade (*n*, %)	Grade ≥3 (*n*, %)
Fatigue	18 (52.9)	11 (32.4)		14 (35.0)	6 (15.0)	
Pain^a^	18 (52.9)			14 (35.0)		
Diarrhoea	14 (41.2)	8 (23.5)	5 (14.7)	18 (45.0)	13 (32.5)	
Ocular symptoms^b^	14 (41.2)	10 (29.4)		14 (35.0)	12 (30.0)	
Cough	10 (29.4)			5 (12.5)		
Dyspnoea	9 (26.5)			9 (22.5)		
Decreased appetite	9 (26.5)	4 (11.8)		13 (32.5)	7 (17.5)	
Nausea	7 (20.6)	5 (14.7)		12 (30.0)	5 (12.5)	
Constipation	6 (17.6)			7 (17.5)		
Vomiting	6 (17.6)			8 (20.0)	5 (12.5)	
Weight loss	4 (11.8)					
Hypotension	5 (14.7)					
Urinary tract infection				6 (15.0)		
Upper respiratory tract infection				5 (12.5)		
Headache				5 (12.5)		
Pruritis				4 (10.0)		
Anaemia				14 (35.0)	6 (15.0)	
Alanine aminotransferase increase				10 (25.0)	6 (15.0)	
Aspartate aminotransferase increase				9 (22.5)	6 (15.0)	
Lipase increase	4 (11.8)		4 (11.8)	7 (17.5)	5 (12.5)	5 (12.5)
Alkaline phosphatase increase				7 (17.5)		
Gamma-glutamyltransferase increase				7 (17.5)		
Hyperglycaemia				5 (12.5)		
Hypoalbuminaemia				5 (12.5)		
Amylase increase				4 (10.0)		
Hypokalaemia				4 (10.0)		

^a^Back pain, musculoskeletal pain, abdominal pain, chest pain, pain not otherwise specified.

^b^Corneal epithelial microcysts, blurred vision, conjunctivitis, keratitis, eye irritation, vitreous haemorrhage, blepharitis, vitreous floaters, increased intraocular pressure.

Based on the 2 DLTs reported at DL 3 (300 mg BID), the MTD was determined to be 250 mg BID. As a result of one patient with grade 1 treatment-related eye toxicity at this DL, the RP2D moving forward in the study was set at DL 200 mg BID.

In the expansion part (using the RP2D), 98% experienced AEs (80% treatment related). The most frequently reported AEs in the expansion part (≥10%) were fatigue (*n* = 14, 35%), diarrhoea (*n* = 18, 45%), ocular symptoms (*n* = 14, 35%), pain (*n* = 14, 35%), decreased appetite (*n* = 13, 33%), nausea (*n* = 12, 30%), dyspnoea (*n* = 9, 23%), vomiting (*n* = 8, 20%), anaemia (*n* = 14, 35%), alanine aminotransferase (ALT) increase (*n* = 10, 25%) and aspartate aminotransferase (AST) increase (*n* = 9, 23%). Toxicity grade ≥3 in ≥10% of patients was increased lipase. TRAEs (≥10%) were diarrhoea (*n* = 13, 33%), ocular symptoms (*n* = 12, 30%), decreased appetite (*n* = 7, 18%), fatigue (*n* = 6, 15%), nausea (*n* = 5, 13%), vomiting (*n* = 5, 13%), ALT increase (*n* = 6, 15%), AST (*n* = 6, 15%), anaemia (*n* = 6, 15%) and lipase increase (*n* = 6, 15%) (Table 3). White blood cell count decrease was observed in two patients and mucositis or stomatitis were observed in only two patients. SAEs and treatment-related SAEs were reported for 40 and 3% of the patients, respectively. Three (8%) patients had AEs leading to discontinuation of the study drug; none were considered treatment related. No AEs with the outcome of death were reported.

In total, ocular side effects were seen in 9 patients (blurred vision, dry eyes, corneal epithelial microcysts, corneal punctate epithelial erosion, punctate epitheliopathy, punctate keratitis, conjunctivitis, increased intraocular pressure). Of them, four had an ocular DLT; two patients were treated with TAS-119 preceding the amended obligatory ophthalmologic examinations (DL 3; 300 mg BID). The ocular toxicity was dose dependent and only seen at doses of 200 mg BID and above.

The 4-week continuous dosing regimen was evaluated in the dose escalation part, in parallel with conducting the expansion part. The continuous dosing schedule has never been initiated in the expansion part based on the preliminary results from the dose escalation part, showing no significant differences between the intermittent and continuous schedule of 200 mg BID. No DLT was observed in 6 patients treated at 200 mg BID in the 4-week continuous dosing regimen.

### PK and PD

**Fig. 1:**
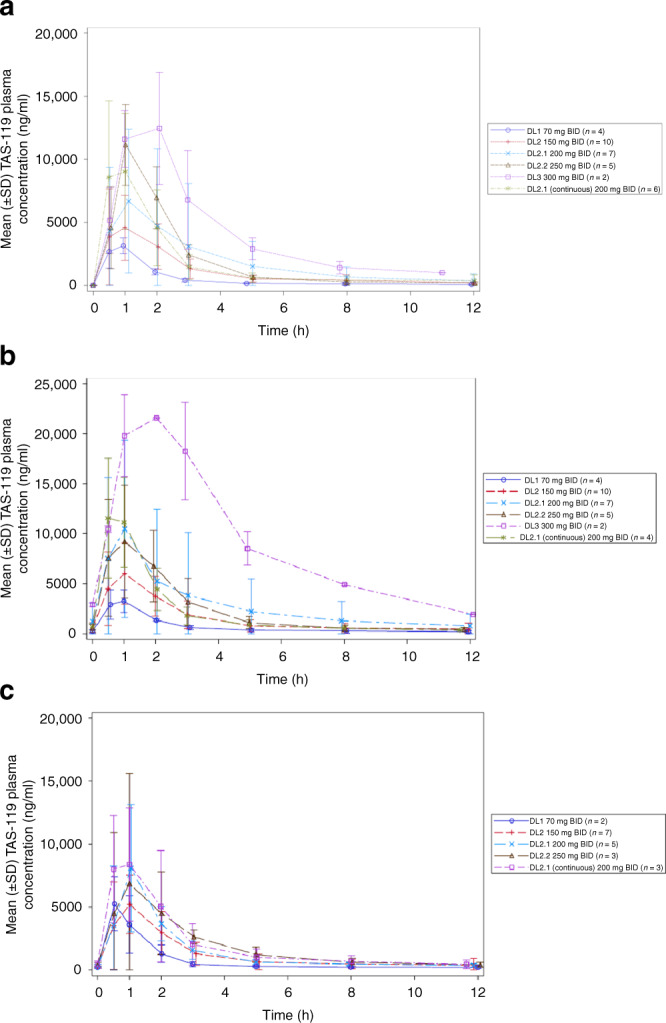
Mean (±SD) plasma concentration versus time curves (linear scales) for TAS-119. **a** Cycle 1 day 1. **b** Cycle 1 day 4. **c** Cycle 1 day 18. A total of 34 patients were evaluable for PK data. In 2 patients, parameters from one pre-dose sample and one day 4 sample, respectively, were unavailable. Parameters collected on a non-predefined day (2 patients) or collected from patients with dose omissions (2 patients) or dose reductions (1 patient) were excluded.

**Fig. 2:**
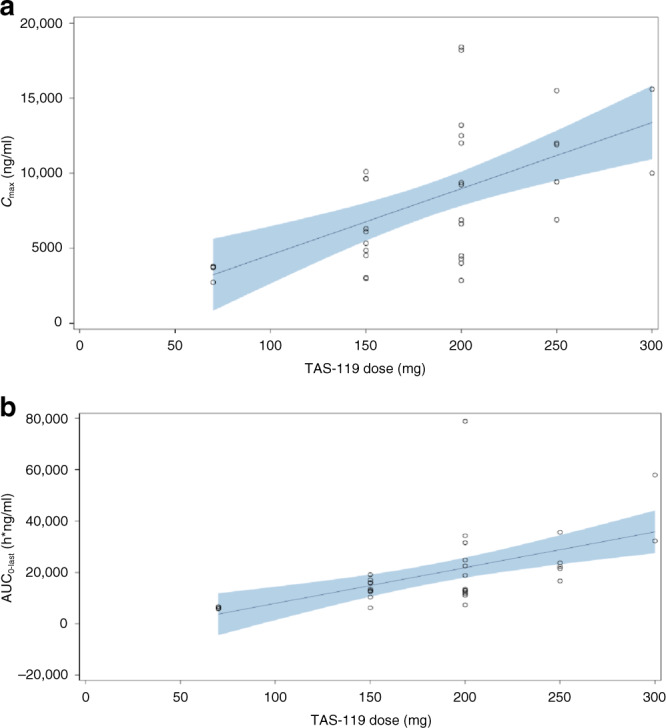
Scatterplots of TAS-119 parameters (*C*_max_ and AUC_0 − last_) versus dose on cycle 1 day 1. **a** *C*_max_ versus dose in cycle 1 day 1. **b** AUC_0-last_ versus dose in cycle 1 day 1. AUC_0 − last_ = area under the plasma concentration–time curve from time 0 to the time point of last observable concentration, *C*_max_ = maximum observed plasma concentration, PK pharmacokinetics. Note: Each symbol represents individual PK parameters. The regression curve was provided by the linear model. The shaded area indicates 90% confidence band.

A total of 34 patients were evaluable for PK data. Mean plasma concentrations over time showed a dose-proportional increase of plasma exposure that did not significantly change after multiple doses on day 4 and on day 18, both being on-treatment days (Fig. 1). One out of 2 patients at DL3 had a relatively high plasma concentration on cycle 1 day 4, but both patients at this DL3 experienced some ocular toxicity. Dose-proportionality analyses by power model, linear model and one-way ANOVA confirmed dose proportionality of TAS-119 PK (Fig. 2).

There was a median *T*_max_ of 1.2 h (range 0.5–2.0 h); blood concentrations declined with a mean half-life between 2.8 and 6.0 h. The main CL_r_ was much lower (median 0.25 L/h, range 0.15–0.31 L/h) than the main oral clearance (CL/F) (median 9.79 L/h, range 2.72–13.94 L/h) in plasma. No trends were observed between dose and urinary PK parameters. The accumulation ratios throughout all DLs were low. At the RP2D level (DL 2.1; 200 mg BID), the mean ratio of AUC_0 − last_ were 1.3 and 1.2 on both days 4 and 18, respectively. This did not significantly differ between the intermittent and continuous 200 mg BID schedule (DL 2.1) with a ratio of 1.2 and 1.1 on days 4 and 18, respectively. The CL/F and *V*_d_/F of TAS-119 were compared among SLCO1B1 gene polymorphism-caused phenotypes (normal/intermediate/low) by ANOVA, and no statistical differences were observed.

**Fig. 3:**
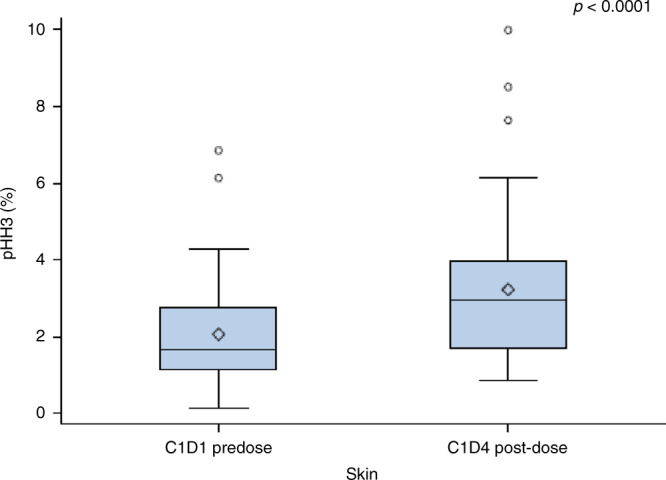
Mean pHH3-positive rates in paired skin samples. Data based on 55 paired skin samples taken prior to TAS-119 administration and after receiving TAS-119 administered on day 4 of cycle 1. The *p* value is based on paired *t* test.

Analyses for target modulation of TAS-119 with respect to mRNA expression were evaluable for 32 patients from the dose escalation part (1 missing sample at DL 200 mg BID and DL 250 mg BID) and showed no significant increase in the expression level. The mean pHH3-positive rate in skin samples, available for 55 paired samples, increased after TAS-119 administration (*p* value <0.0001; Fig. 3). The mean pHH3 rate did not significantly change in paired tumour samples, although the sample size was very small (eight paired tumour samples).

### Antitumour activity

In the dose escalation part, no complete response (CR) or partial response (PR) was observed. Fourteen (41%) patients had stable disease as best response: 6 patients at DL 2 (150 mg BID), 4 patients at DL 2.1 (200 mg BID), and 4 patients at DL 2.1 (200 mg BID continuous schedule). One patient with an epithelial mesothelioma at DL 2 (150 mg BID) had an unconfirmed PR at cycle 4.

In the expansion part of the study, no confirmed CR or PR were observed. Fourteen (35%) patients had stable disease (5 SCLC, 5 *MYC*-amp/B-cat mutation-positive tumours, 1 breast cancer and 2 basket cohort patients (1 mesothelioma, 1 colorectal cancer). Based on the observed response in the dose escalation part, additional mesothelioma patients were enrolled in the basket cohort of the trial (Table 1). No additional PRs or confirmed CRs were observed in any of these patients.

The target ORR of 20% (or ≥10% for patients with *MYC* amplification or β-catenin mutation) was not met in the expansion part, and therefore enrolment in the study was discontinued and the extension part of the expansion part has not been conducted.

## Discussion

Here we report on a first-in-human phase 1 study with TAS-119, an oral selective AurA kinase inhibitor. TAS-119 was largely well tolerated, with a low frequency of treatment discontinuation due to AEs. Grade ≥3 toxicities included diarrhoea (5/74 patients) and increased lipase without symptoms of pancreatitis (9/74 patients).

Diarrhoea, decreased appetite and increases in AST and/or ALT levels were expected AEs based on pre-clinical evaluation of TAS-119 and based on what has been seen in other AurA inhibitor trials.^12^ Increased lipase levels were not expected. It is remarkable that the most commonly observed grade 3 drug-related toxicities in most other AurA inhibitor trials, such as (febrile) neutropenia, thrombocytopenia, anaemia and stomatitis,^11,12,14^ were only very infrequently observed in this study. These side effects have hindered clinical development and application of various other AurA inhibitors but were not significantly influencing patient’s well-being or safety in this study. This observation can likewise be explained by either the different cross-inhibition pattern of other kinases with TAS-119 in comparison to other selective AurA inhibitors or the use of an intermittent administration schedule.

Ocular AEs and DLTs led to the selection of 200 mg BID as the RP2D administered 4 days on/3 days off, every 3 out of 4 weeks, and therefore this dose and schedule was used in the expansion part of this trial.

The ocular toxicity in 38% of the patients throughout the various DLs is of particular concern. Ocular toxicity that became clear as a result of a decrease in visual acuity, mainly related to problems localised within the cornea, led to the incorporation of routine ophthalmologic examinations in subsequent patients in the trial. Microscopic findings in the pre-clinical high-dose toxicity study in animals showed degeneration and regeneration or hyperplasia of epithelial tissue, with considerable individual variation between animals and, only in dogs, involvement of the epithelium of the eyes. Expert review determined that it was plausible that the corneal events appeared to be due to excretion of TAS-119 into the tear film (not measured), with secondary direct irritation, and/or the effects of the agent on rapidly growing cells in the corneal basal epithelium. The events were dose dependent, temporary and resolved with cessation of treatment. The need to discontinue treatment because of these events was rare.

The observed corneal toxicity may be caused by a direct off-target toxic effect on the epithelium of the cornea, as well as an on-target effect by influencing the normal epithelial–mesenchymal transition (EMT) process of the corneal epithelium. EMT plays a role in the self-renewal and homoeostasis of the cornea^20^ and active AurA is associated with mitogen-activated protein kinase pathway-induced EMT.^21^ As a consequence of inhibiting AurA, it is conceivable that epithelial markers can be upregulated (reverse EMT).^21^ Considering this mechanism, one could have expected that corneal toxicity would have occurred in other trials with AurA kinase inhibitors. However, and to the best of our knowledge, ocular toxicity has not been described in other clinical trials with AurA kinase inhibitors,^2,10–12^ except for the new generation AurA inhibitor LY3295668, where corneal deposits were described without further specification.^22^ It should be taken into consideration that inhibition of other kinases by TAS-119 might also play a role in the ocular toxicity. The exact underlying mechanism(s) of ocular toxicity associated with TAS-119 require further investigation.

Target modulation of TAS-119 was shown in 55 paired skin biopsies with an increase in pHH3-positive cells after administration (Fig. 3). This was not confirmed in tumour biopsies; however, the sample size was very small (eight paired tumour samples) and therefore it is not feasible to draw firm conclusions.

TAS-119 demonstrated limited antitumour activity as single agent. Pre-clinical work indicated more growth inhibitory activity of AurA inhibitors on cancer cells with *MYC* oncogene amplifications and/or mutations in the Wnt/β-catenin pathway with decreased MYC protein expression in the presence of AurA kinase inhibition,^16,17^ but this could not be confirmed clinically.^23^ In conclusion, these genetic aberrations do not seem to have potential as a predictive biomarker for TAS-119. Because the predefined target of an ORR of 20% (or ≥10% for patients with *MYC* amplification or β-catenin mutation) was not met in the expansion part of this trial, enrolment in the study was not extended for more patients with *MYC* amplification or β-catenin mutation.

In conclusion, a twice-daily 200 mg dose in an intermittent schedule (4 days on 3 days off, 3 out of 4 weeks) was established as the dose and schedule for further activity testing of TAS-119. The RP2D was not merely determined by the overall toxicity profile but ocular toxicity was crucial in the decision. Evidence for target modulation was acquired but the observed antitumour activity of TAS-119 was disappointing. Although the observed ocular toxicity merits attention, the overall safety profile of TAS-119 seems to stand out when compared to that of other AurA inhibitors. These data support the further investigation of TAS-119 in pre-clinical combination trials to look for potential synergistic effects as well as in early clinical trials in combination with drugs influencing cell cycle processes.

## Supplementary information

Supplementary Material

## Data Availability

All data supporting the results in this manuscript are available at Taiho Oncology.
