# Supplementary material for: A first-in-human phase 1 and pharmacological study of TAS-119, a novel selective Aurora A kinase inhibitor in patients with advanced solid tumours
Source: Br J Cancer. 2020 Oct 6;124(2):391–8. doi: 10.1038/s41416-020-01100-3 (PMC7852567; doi:10.1038/s41416-020-01100-3)
Supplement: Supplementary file 1 — Supplementary Material [file 41416_2020_1100_MOESM1_ESM.docx]

**Supplementary information:**

- Supplementary table 1.xlxs

Table containing information on patient disposition in the dose escalation phase

**Supplementary table 1. Patient disposition in the dose escalation phase**

|  | DL 1 | DL 2 | DL 2.1 | DL 2.1 | DL 2.2 | DL 3 |
| --- | --- | --- | --- | --- | --- | --- |
|  | 70 mg BID | 150 mg BID | 200 mg BID | 200 mg BID cont. | 250 mg BID | 300 mg BID |
|  | n (%) | n (%) | n (%) | n (%) | n (%) | n (%) |
| treated patients | 4 | 10 | 7 | 6 | 5 | 2 |
| DLT evaluable patients | 3 (75.0) | 10 (100.0) | 6 (85.7) | 5 (83.3) | 4 (80.0) | 2 (100.0) |
| DLT occurence | 0 | 1 | 1 | 0 | 1 | 2 |
| The DLT Population comprised 30 patients; 4 (11.8%) were excluded because they received | | | | | |  |
| < 80% of the planned dose in Cycle 1 without a DLT. | | | |  |  |  |
| DLT = dose-limiting toxicity, cont. = continuous schedule | | | |  |  |  |

- Supplementary text.docx

**Inclusion and exclusion criteria of the trial**

**Inclusion Criteria**

1. Is a male or female ≥18 years of age, who has provided written informed consent

2. Has histologically or cytologically confirmed advanced, unresectable, and/or metastatic solid tumour(s) for which the patient has no available therapy likely to provide clinical benefit.

3. Has Eastern Cooperative Oncology Group (ECOG) performance status 0 or 1 on Cycle 1, Day 1.

4. Must have available an archival formalin-free, paraffin-embedded (FFPE) tumour sample

to be provided upon Sponsor request.

5. In the Expansion Phase: patients should be willing to undergo optional core tumour

biopsy procedure (on or before Day 1, Cycle 1) and on Day 4, Cycle 1 (6 hours ± 2 hours

postdose) if, in the judgment of the investigator, it is considered clinically safe and

appropriate to do so. This requirement is optional but preferred for patients in Dose

Escalation.

6. Should be willing to undergo pretreatment sampling of non-tumour surrogate tissue for

pharmacodynamic assessments (ie, a 3 mm punch biopsy of skin from either the arm or

the back of the scalp) if, in the judgment of the investigator, it is considered clinically

safe and appropriate to do so.

7. Is able to take medications orally (eg, no feeding tube).

8. Has adequate organ function as defined by the following criteria:

a. Aspartate aminotransferase (AST/serum glutamic oxaloacetic transaminase [SGOT])

and alanine aminotransferase (ALT/serum glutamic pyruvic transaminase [SGPT])

≤ 3.0 × upper limit of normal (ULN).

b. Total serum bilirubin within normal limits. Patients with Gilbert's Syndrome could be

considered eligible if bilirubin is ≥ 1 × ULN, but serum bilirubin ratio of

unconjugated/conjugated is greater than 1 or most of serum bilirubin fraction should

be unconjugated.

c. Absolute neutrophil count of ≥ 1,500/mm3 (ie, ≥ 1.5 × 109/L by International Units

[IU]).

d. Platelet count ≥ 100,000/mm3 (IU: ≥ 100 × 109/L). Transfusion of whole blood,

platelets, or RBCs is prohibited within 1 month prior to initiation of study treatment.

e. Hemoglobin value of ≥ 9.0 g/dL.

f. Total serum creatinine of ≤ 1.5 × ULN.

g. Serum albumin ≥ 2.5 g/dL.

9. Women of childbearing potential must have a negative pregnancy test (urine or serum)

within 7 days prior to starting the study drug. Both males and females and must agree to

use effective birth control during the study [prior to the first dose and for 6 months after

the last dose if conception is possible during this interval] if conception is possible during

this interval. Female patients are considered to not be of childbearing potential if they

have a history of tubal ligation or hysterectomy or are post-menopausal with a minimum

of 1 year without menses.

10. Is willing and able to comply with scheduled visits and study procedures.

**Exclusion Criteria**

1. Has received prior treatment with TAS-119.

2. Has received treatment with any of the following within the specified time frame prior to

study drug administration:

a. Major surgery within prior 4 weeks (the surgical incision should be fully healed prior

to study drug administration).

b. Radiotherapy for extended field within 4 weeks prior to study drug administration or

limited field radiotherapy within 2 weeks prior to study drug administration.

c. Previous cytotoxic chemotherapy for advanced or metastatic solid tumors consisting

of more than 5 different regimens in total.

d. Any anticancer therapy within 3 weeks prior to study drug administration (mitomycin

within prior 5 weeks). Patients with metastatic prostate cancer receiving luteinizing

hormone-releasing (LHRH) analogs will be eligible.

e. Any investigational agent received either concurrently or within the last 30 days.

f. Transfusion of whole blood, platelets, or packed red blood cells is prohibited within 1

month prior to initiation of study treatment.*

3. Has a serious illness or medical condition(s) including, but not limited to, the following:

a. Known brain metastasis unless the lesions have been previously treated with surgery

or radiotherapy, and have been stable off steroids for ≥ 2 months.

b. Known leptomeningeal metastasis.

c. Known acute systemic infection.

d. Myocardial infarction, severe/unstable angina, symptomatic congestive heart failure

(New York Heart Association [NYHA] class III or IV) within the previous 6 months;

if > 6 months cardiac function must be within normal limits (ejection fraction ≥ 50%)

and the patient must be free of cardiac-related symptoms.

e. Chronic nausea, vomiting, or diarrhoea, considered to be clinically significant in the

opinion of the Investigator.

f. Known human immunodeficiency virus (HIV) or acquired immunodeficiency

syndrome (AIDS)-related illness, or a history of serum positivity to hepatitis B or C.

g. Other severe acute or chronic medical or psychiatric condition or laboratory

abnormality that may increase the risk associated with study participation or study

drug administration, or may interfere with the interpretation of study results, and in

the judgment of the Investigator would make the patient inappropriate for entry into

this study.

4. Has known hypersensitivity to TAS-119 or any of its components.

5. Is a pregnant or lactating female.
